# Metal-Ion-Free Preparation of κ-Carrageenan/Cellulose Hydrogel Beads Using an Ionic Liquid Mixture for Effective Cationic Dye Removal

**DOI:** 10.3390/gels11080596

**Published:** 2025-08-01

**Authors:** Dojin Kim, Dong Han Kim, Jeong Eun Cha, Saerom Park, Sang Hyun Lee

**Affiliations:** 1 Advanced Materials Program, Department of Biological Engineering, Konkuk University, Seoul 05029, Republic of Korea; dojin135@naver.com (D.K.); edike1211@naver.com (D.H.K.); cje0430@konkuk.ac.kr (J.E.C.); 2 Department of Biological Engineering, Konkuk University, Seoul 05029, Republic of Korea

**Keywords:** κ-carrageenan, cellulose, metal-ion-free, hydrogel bead, adsorption, crystal violet

## Abstract

A metal-ion-free method was developed to prepare κ-carrageenan/cellulose hydrogel beads for efficient cationic dye removal. The beads were fabricated using a mixture of 1-ethyl-3-methylimidazolium acetate and N,N-dimethylformamide as the solvent system, followed by aqueous ethanol-induced phase separation. This process eliminated the need for metal-ion crosslinkers, which typically neutralize anionic sulfate groups in κ-carrageenan, thereby preserving a high density of accessible binding sites. The resulting beads formed robust interpenetrating polymer networks. The initial swelling ratio reached up to 28.3 g/g, and even after drying, the adsorption capacity remained over 50% of the original. The maximum adsorption capacity for crystal violet was 241 mg/g, increasing proportionally with κ-carrageenan content due to the higher surface concentration of anionic sulfate groups. Kinetic and isotherm analyses revealed pseudo-second-order and Langmuir-type monolayer adsorption, respectively, while thermodynamic parameters indicated that the process was spontaneous and exothermic. The beads retained structural integrity and adsorption performance across pH 3–9 and maintained over 90% of their capacity after five reuse cycles. These findings demonstrate that κ-carrageenan/cellulose hydrogel beads prepared via a metal-ion-free strategy offer a sustainable and effective platform for cationic dye removal from wastewater, with potential for heavy metal ion adsorption.

## 1. Introduction

Hydrogels are three-dimensional (3D) polymeric networks composed of hydrophilic polymers that can absorb large amounts of water without dissolving [1,2]. They are classified based on their origin, preparation methods, conformational structure, and physicochemical properties, which can be tailored for diverse applications [1,3]. Depending on their source, hydrogels may contain ionic or neutral functional groups—for example, alginate (anionic), chitosan (cationic), collagen (amphiphilic), and agarose (neutral). Their responsiveness to environmental stimuli such as temperature, pH, ionic strength, and electric or magnetic fields enables their use in agriculture, food additives, wastewater treatment, pharmaceuticals, and biomedicine [3,4,5]. Among these, natural polymer-based hydrogels have received growing interest owing to their low cost, biodegradability, and biocompatibility [6]. However, such hydrogels often lack sufficient mechanical strength and the functional moieties required for specific interactions, including ionic adsorption. To address these limitations, recent studies have introduced complementary biopolymer combinations or functionalized networks with groups like sulfate, thereby improving mechanical properties and application-specific performance [7,8,9,10,11].

Carrageenans are sulfated polysaccharides derived from red algae (Rhodophyceae) that are widely used as gelling, thickening, and stabilizing agents in the food industry [12,13,14]. They consist of linear galactan chains containing 15–40% sulfate groups and are classified into κ-, ι-, and λ-types based on their structure and gelation behavior [12,14,15]. κ-Carrageenan forms rigid and brittle gels, while ι-carrageenan generates softer and more elastic networks [15,16]. Gelation typically requires crosslinking with divalent or monovalent cations such as Ca^2+^ or K^+^ [17,18]. Importantly, κ-carrageenan features sulfate (–OSO_3_^−^) groups with very low pKa values (<2), imparting a high density of negative charges that facilitate electrostatic interactions with cationic species. Compared to other biopolymers such as alginate, the carboxyl groups of which have higher pKa values (~3.5), carrageenan is especially attractive for applications in drug delivery, cell encapsulation, and wastewater treatment [19]. However, metal-ion-induced gelation can reduce the availability of sulfate groups for adsorption by neutralizing anionic sites, which limits the performance of conventional carrageenan hydrogels.

Industrial processes such as textile dyeing, papermaking, cosmetics production, and leather tanning discharge large quantities of dyes and heavy metals into wastewater. These contaminants reduce water clarity, increase chemical oxygen demand, and exhibit acute toxicity toward aquatic life [20,21]. The associated environmental and health risks necessitate the development of effective treatment technologies. Various approaches have been employed, including electrochemical oxidation, catalytic degradation, biological treatment, membrane filtration, and adsorption [22,23]. Among these, adsorption is widely favored due to its simplicity, cost-effectiveness, and broad applicability [24]. Carrageenan-based hydrogels offer considerable potential as adsorbents for cationic dyes because of their dense sulfate functionalities. However, conventional fabrication methods that require metal-ion crosslinking compromise adsorption capacity by decreasing the number of accessible sulfate groups. Thus, strategies that eliminate the need for such crosslinkers are essential to enhance both functional group retention and environmental compatibility. Avoiding metal-ion crosslinkers also reduces contamination risk and simplifies processing, supporting a more sustainable and economical fabrication route.

Ionic liquids have emerged as effective solvents for processing natural polymers into functional hydrogels without requiring metal ions. Other green solvents such as deep eutectic solvents and physical methods like freeze–thaw gelation have also been reported but often lack sufficient polymer solubility or functional group accessibility. In addition, recent improvements in the synthetic process and reductions in raw material costs, especially for imidazole-based ionic liquids, have enhanced the economic feasibility of their large scale use. For carrageenan, ionic liquids enable improved water uptake and compatibility with other biopolymers [25,26,27]. Nevertheless, hydrogels based solely on κ-carrageenan are still limited by poor mechanical stability, low reusability, and sensitivity to acidic conditions [28]. In contrast, cellulose-based hydrogels prepared using ionic liquids exhibit excellent structural integrity and durability. However, cellulose contains only neutral hydroxyl groups and lacks the ionic functionalities required for effective electrostatic adsorption. A viable approach is to combine cellulose with κ-carrageenan to create composite hydrogels that integrate mechanical strength and functional adsorption sites. In our previous work, we developed cellulose/κ-carrageenan/TiO_2_ composite films capable of adsorbing and photodegrading methylene blue [29]. Although that system achieved good performance, it relied on a film-type geometry that required different solvent and antisolvent systems for fabrication. In contrast, the present study focuses on the development of κ-carrageenan/cellulose hydrogel beads, which offer improved sulfate group accessibility, reusability, and fabrication simplicity, making them well-suited for efficient cationic dye removal from aqueous environments. To systematically explore the effects of biopolymer composition on dye adsorption behavior and reusability, we maintained a total polymer concentration of 7% and varied the κ-carrageenan-to-cellulose ratio in the bead formulation. The bead-type geometry was selected to enhance surface accessibility and facilitate recovery in aqueous applications.

In this study, κ-carrageenan/cellulose hydrogel beads were prepared using the ionic liquid 1-ethyl-3-methylimidazolium acetate ([Emim][Ac]), with N,N-dimethylformamide (DMF) as a co-solvent. [Emim][Ac] has also become more economically viable due to recent improvements in its production process and the reduced cost of its starting materials. No metal ions such as K^+^ or Ca^2+^ were employed during gelation. The swelling behavior of the hydrogel beads was first characterized through both initial and re-swelling ratio measurements to assess water uptake and structural stability after drying. Adsorption performance was evaluated using crystal violet (CV) as a model cationic dye under various conditions, including different contact times, initial dye concentrations, and pH levels. The adsorption kinetics, isotherm models, and thermodynamic properties were systematically analyzed. In addition, the pH stability of the hydrogel beads was assessed, and their reusability was evaluated over multiple adsorption–desorption cycles. This study presents a novel approach for synthesizing κ-carrageenan/cellulose hydrogel beads without the use of metal-ion crosslinkers, utilizing ionic liquids to preserve sulfate group functionality and enable efficient adsorption of cationic dyes.

## 2. Results and Discussion

### 2.1. Characteristics of κ-Carrageenan/Cellulose Hydrogel Beads

In this study, κ-carrageenan/cellulose hydrogel beads containing various concentrations of κ-carrageenan were prepared, maintaining the total concentration of biopolymers (cellulose and κ-carrageenan) at 7% in the gel-forming solution. Both κ-carrageenan and cellulose were simultaneously dissolved in [Emim][Ac], but the high viscosity of the solution posed challenges in forming spherical hydrogel beads, thus necessitating the addition of a co-solvent. DMF and dimethyl sulfoxide (DMSO) were used to reduce the viscosity of [Emim][Ac]-based cellulose solutions. As confirmed in our previous study [30], cellulose remained fully dissolved within the tested DMF range, indicating no significant loss in solubility under these conditions. DMF was more effective than DMSO in reducing viscosity. Therefore, an [Emim][Ac]/DMF mixture (65/35 *v*/*v*) was selected as the dissolution solvent in this study. This ratio was chosen based on preliminary tests, where various DMF concentrations were evaluated, and 65/35 (*v*/*v*) provided the best viscosity for forming spherical beads without tailing. In addition, the antisolvent used to regenerate the dissolved biopolymers significantly influenced the shape of the hydrogel beads. When water, a commonly used antisolvent, was used, the resulting beads exhibited entrapped bubbles and poor structural integrity, often floating on the water surface. To overcome this issue, 20% ethanol was used as the antisolvent instead of pure water [29]. Generally, carrageenan requires metal ions such as K^+^ or Ca^2+^ to gel; however, in this study, robust κ-carrageenan/cellulose hydrogels were formed without any metal ions. This is likely due to rapid aqueous ethanol-induced phase separation, which precipitates both polymers into a physically crosslinked 3D network reinforced by cellulose hydrogen bonding and partial recrystallization [29]. Additionally, electrostatic and hydrogen bonding interactions between carrageenan sulfate groups (–OSO_3_^−^) and cellulose hydroxyls (–OH) contribute to the formation of an interpenetrating network with enhanced mechanical stability.

**Figure 1:**
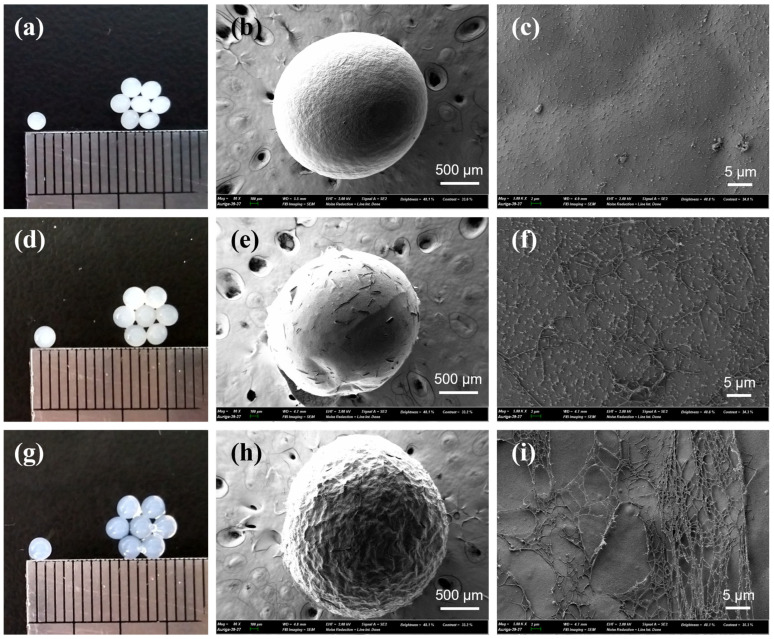
Photographic and SEM images of cellulose-only (7%) hydrogel beads (**a**–**c**), κ-carrageenan/cellulose (2%/5%) hydrogel beads (**d**–**f**), and κ-carrageenan/cellulose (4%/3%) hydrogel beads (**g**–**i**). The combined concentration of κ-carrageenan and cellulose in the gel-forming solution (65/35% *v/v* [Emim][Ac]/DMF) was fixed at 7%.

Figure 1 shows photographic and scanning electron microscopy (SEM) images of cellulose and κ-carrageenan/cellulose hydrogel beads. In all cases, the beads exhibit a uniformly spherical shape. As the κ-carrageenan content increases, the beads become more transparent, as observed in Figure 1a,d,g. Simultaneously, the surface texture changes from a smooth, cellulose-dominated structure (Figure 1c) to a rough, fibrous network (Figure 1f,i). This morphological change is attributed to ethanol-induced phase separation, during which both polymers are precipitated into a physically crosslinked 3D network. At lower cellulose contents, the reduced formation of crystalline domains may weaken the internal matrix, allowing κ-carrageenan chains to accumulate at the surface and form fibrous structures.

The bead interior may be reinforced by extensive cellulose–cellulose hydrogen bonding and partial recrystallization during the antisolvent-induced gelation process. Additionally, electrostatic and hydrogen bonding interactions between the sulfate groups (–OSO_3_^−^) of κ-carrageenan and the hydroxyl groups (–OH) of cellulose may contribute to the formation of a uniform interpenetrating network, which in turn reduces light scattering and improves optical transparency. To gain further insight into the internal structure associated with transparency changes upon varying the κ-carrageenan content, films composed of pure cellulose, κ-carrageenan/cellulose (2%/5%), and κ-carrageenan/cellulose (4%/3%) were prepared and analyzed for their visible light absorbance characteristics (Figure S1). As the κ-carrageenan content increased, a marked decrease in overall absorbance was observed across the 400–1000 nm range, indicating enhanced optical transparency. This trend confirms that higher κ-carrageenan content contributes to improved transparency in the corresponding hydrogel beads, consistent with reduced light scattering due to a more homogeneous interpenetrating network. A similar behavior has been reported for cellulose-derivative/κ-carrageenan double-network hydrogels [31].

Table 1 presents the wet bead size and dried weight of the κ-carrageenan/cellulose beads prepared with various κ-carrageenan ratios. As the κ-carrageenan fraction increased, the wet bead diameter increased, whereas the dried bead mass decreased. This trend is likely due to the highly hydrophilic nature of the sulfate-rich carrageenan network, which enables extensive water uptake during swelling. However, its lower density and reduced crystallinity result in less solid residue after drying. In contrast, cellulose-rich beads showed higher dried weights but exhibited significantly less swelling compared to carrageenan-rich beads. As a result, carrageenan-rich beads are likely to form a more open and porous structure, which facilitates high water uptake and enhances the accessibility of internal adsorption sites. These structural characteristics are consistent with the enhanced cationic dye adsorption capacity observed in subsequent experiments.

### 2.2. Swelling Property of κ-Carrageenan/Cellulose Hydrogel Beads

**Figure 2:**
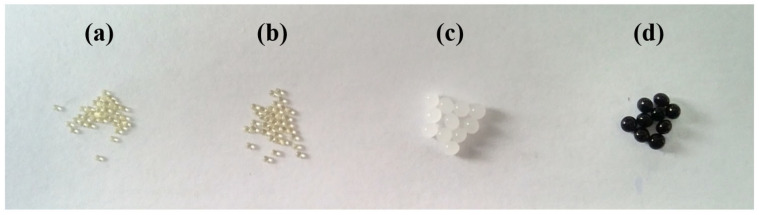
Photographic images of κ-carrageenan/cellulose (4%/3%) hydrogel beads (CN4C3): (**a**) dried beads, (**b**) re-swelled beads after drying, (**c**) initial hydrogel beads, and (**d**) hydrogel beads after CV adsorption.

κ-Carrageenan chains bear negatively charged sulfate groups, making them more hydrophilic than cellulose. As a result, carrageenan-rich beads exhibit higher swelling ratios, which may contribute to enhanced uptake of cationic dyes. Figure 2 compares freshly prepared CN4C3 hydrogel beads (Figure 2c), the same beads after drying (Figure 2a), and the dried beads after re-swelling in water for 48 h (Figure 2b). Upon drying, the hydrogel network collapsed as the polymer chains approached one another and formed irreversible hydrogen bonds. This led to structural densification and a substantial reduction in bead volume (Figure 2a). When re-immersed in water, the collapsed network did not fully reopen, and the re-swollen beads remained smaller than their original size (Figure 2b). This irreversible shrinkage results in a denser internal matrix with reduced pore accessibility, which may localize dye-binding sites near the surface and affect both the rate and extent of dye adsorption.

**Figure 3:**
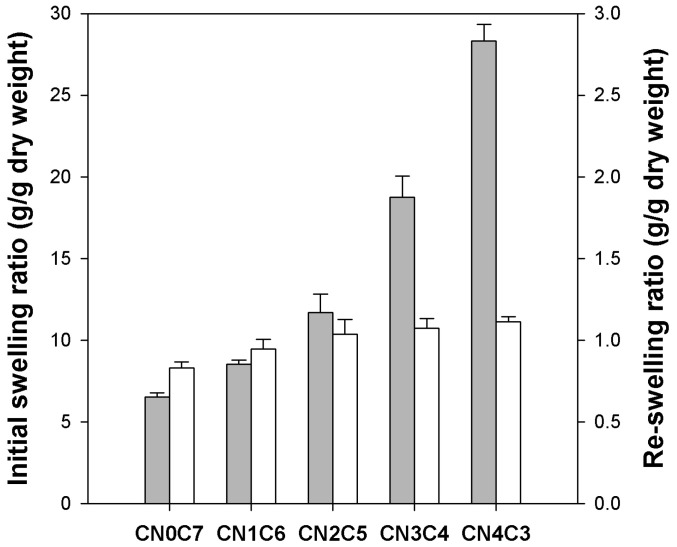
Swelling ratio of initial κ-carrageenan/cellulose hydrogel beads (gray bars) and re-swelling ratio of dried κ-carrageenan/cellulose beads (white bars).

The initial swelling ratio of freshly prepared beads was measured to examine the influence of κ-carrageenan content, as illustrated in Figure 3 (gray bars). The pure cellulose beads exhibited an initial swelling ratio of 6.5 g/g dry weight. A significant increase in swelling ratio was observed with higher κ-carrageenan contents, reaching 28.3 g/g dry weight for CN4C3. After drying, the re-swelling ratio of the beads was determined to evaluate their water uptake capacity upon rehydration (white bars in Figure 3). Upon rehydration, dried cellulose beads showed a re-swelling ratio of 0.83 g/g dry weight, while dried CN4C3 beads reached 1.11 g/g dry weight. The swelling capacity of all beads was substantially reduced after drying, with a greater reduction observed in those with higher κ-carrageenan contents. The swelling capacity of pure cellulose beads decreased by approximately 87%, while that of CN4C3 beads decreased by nearly 96%. The increase in initial swelling with higher κ-carrageenan content can be attributed to the high hydrophilicity of the sulfate groups. These groups promote water uptake and facilitate expansion of the hydrogel network [32]. The substantial reduction in swelling capacity after drying is attributed to the collapse and densification of the polymer network. During drying, the polymer chains moved closer together and formed irreversible hydrogen bonds, thereby hindering full reopening of the pores [33]. This collapse was more pronounced in beads with higher κ-carrageenan contents, as their initially large pore volume underwent greater compaction during drying.

### 2.3. Adsorption Property of κ-Carrageenan/Cellulose Hydrogel Beads for CV

**Figure 4:**
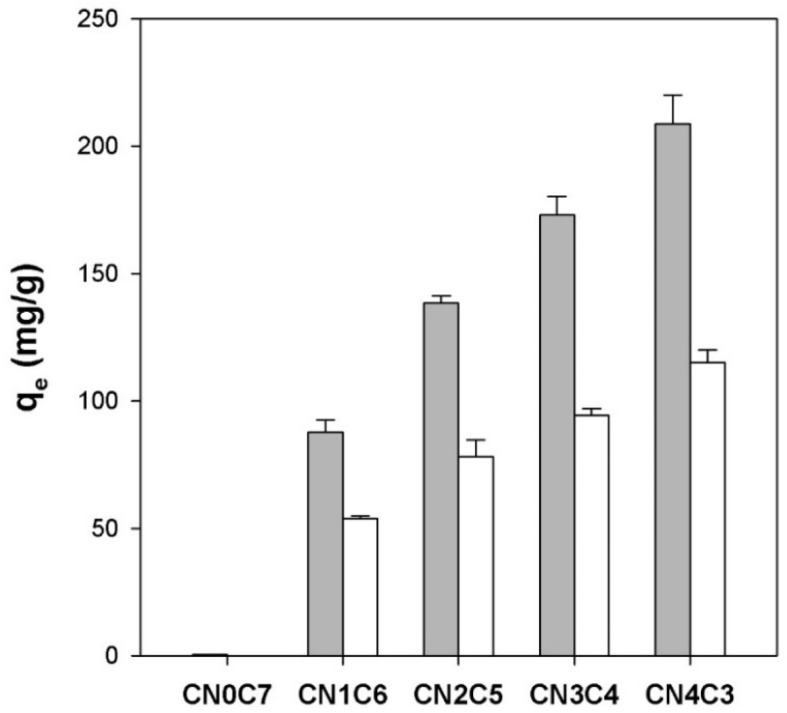
Equilibrium adsorption capacity (q_e_) for CV of initial κ-carrageenan/cellulose hydrogel beads (gray bars) and dried κ-carrageenan/cellulose beads (white bars). Beads were incubated in a 50 mg/L CV solution (pH 7.0) for 24 h at 25 °C.

Figure 4 (gray bars) shows the equilibrium adsorption capacity (q_e_) of freshly prepared carrageenan/cellulose hydrogel beads for CV. The pure cellulose beads (CN0C7) showed negligible uptake (q_e_ = 0.5 mg/g). In contrast, carrageenan-containing beads exhibited a near-linear increase in q_e_ with increasing carrageenan fraction (*X*_CN_), from 88 mg/g at *X*_CN_ = 0.14 (CN1C6) to 209 mg/g at *X*_CN_ = 0.57 (CN4C3). This trend followed a linear regression:

q_e_ = 278.3 × *X*_CN_ + 52.6   (r^2^ = 0.991, *n* = 4) (1)

The dramatic enhancement of CV adsorption with increasing κ-carrageenan content reflects the growing density of negatively charged sulfate sites, which bind cationic CV through electrostatic attraction and hydrogen bonding. Pure cellulose beads lack such anionic sites and thus show almost no uptake. The observed linear dependence of q_e_ on the carrageenan fraction highlights the dominant role of sulfate group concentration in determining the adsorption capacity for CV.

To investigate the adsorption of CV on CN4C3, a film with the same composition was prepared, and the FT-IR spectra of the film was analyzed before and after CV adsorption. As shown in Figure S2, no significant changes were observed at 931, 1017, or 1156 cm^−1^ after CV adsorption, indicating minimal interaction at the corresponding functional groups. These bands correspond to 3,6-anhydro-D-galactose bonds, glucoside linkages, and C-O stretching, respectively [34,35]. However, after CV adsorption, a new band appeared at 1592 cm^−1^, which corresponds to the aromatic C=C stretching of CV [36]. In addition, the band at 847 cm^−1^, assigned to sulfate (–OSO_3_^−^) stretching at the C-4 position of galactose, was shifted to 844 cm^−1^. These results suggest that CV was absorbed onto the film via electrostatic interactions with the sulfate groups of κ-carrageenan by electrostatic attraction [37].

The CV adsorption capacity of the dried beads was also measured (white bars in Figure 4) to assess the effect of network collapse. For pure cellulose beads (CN0C7), the q_e_ value was reduced from 0.5 mg/g before drying to 0.1 mg/g after drying. For CN1C6, q_e_ decreased from 87.8 to 53.8 mg/g, retaining approximately 61% of its initial capacity. Similarly, CN2C5 decreased from 138.3 to 78.1 mg/g, CN3C4 from 173.0 to 94.3 mg/g, and CN4C3 from 208.8 to 115.1 mg/g, each retaining approximately 55% of their original q_e_. Although the swelling ratio fell by over 90% after drying (Figure 3), adsorption capacity remained relatively high. This resilience may be due to the possible migration of carrageenan chains toward the bead surface during drying, which could help preserve a dense layer of anionic sulfate sites. However, this remains a hypothetical explanation requiring further investigation.

#### 2.3.1. Kinetic Study of CV Adsorption on κ-Carrageenan/Cellulose Hydrogel Beads

**Figure 5:**
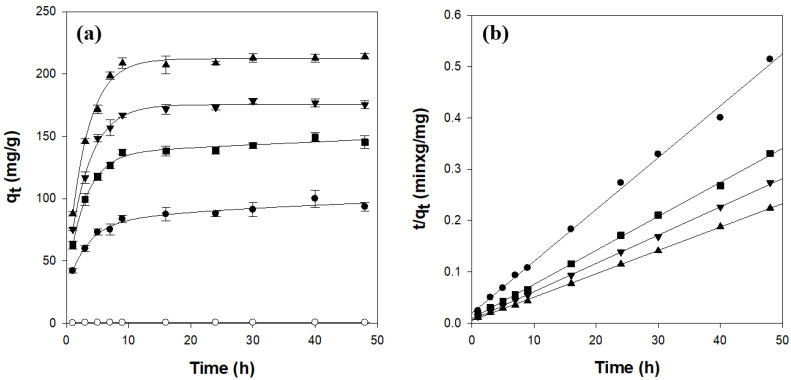
Adsorption kinetics (**a**) and pseudo-second-order model fitting (**b**) for CV adsorption on κ-carrageenan/cellulose hydrogel beads. Beads were incubated in a 50 mg/L CV solution (pH 7.0) at 25 °C. ○: CN0C7, ●: CN1C6, ◼: CN2C5, ▼: CN3C4, ▲: CN4C3.

Figure 5a presents the time-dependent adsorption of CV onto κ-carrageenan/cellulose hydrogel beads in a 50 mg/L solution (pH 7, 25 °C). The pure cellulose beads (CN0C7) adsorbed only 0.5 mg/g of CV and reached equilibrium in approximately 8 h. In contrast, all carrageenan-containing beads required approximately 10 h to reach equilibrium, with equilibrium loadings increasing with κ-carrageenan content.

A pseudo-second-order kinetic model, which best fitted the experimental data, was employed to investigate the CV adsorption kinetics. The model is expressed by the following equation [38]:

(2) $$\frac{t}{q_{t}}=\frac{1}{k_{2}q_{e}^{2}}+\frac{t}{q_{e}}$$

where k_2_ (g/mg/h) is the rate constant of the pseudo-second-order kinetic model; q_t_ (mg/g) is the amount of CV adsorbed at time t (h); and q_e_ (mg/g) is the theoretical adsorption capacity, which can be calculated from the plot of t/q_t_ vs. t. As shown in Table 2 and Figure 5b, the κ-carrageenan/cellulose hydrogel beads exhibited a straight line with high correlation coefficient values (r^2^ > 0.997) when represented in plots of t/q_t_ against t. In addition, the experimental q_e_ values were highly consistent with the theoretically calculated ones. These results confirmed that CV adsorption onto the κ-carrageenan/cellulose hydrogel beads followed pseudo-second-order kinetics. Notably, k_2_ decreased slightly with increasing κ-carrageenan content, indicating that although the adsorption capacity of beads increased, the rate of dye uptake slowed marginally, likely because of longer diffusion paths in the more swollen network. A comparison of the initial adsorption rates, defined as k_2_q_e_^2^, with the carrageenan fraction (*X*_CN_) revealed a clear linear relationship:

k_2_q_e_^2^ = 329,906 × *X*_CN_ + 10,162 (r^2^ = 0.988, *n* = 4) (3)

This strong correlation indicates that the initial dye uptake rate increases proportionally with the density of the anionic sulfate sites provided by κ-carrageenan.

**Figure 6:**
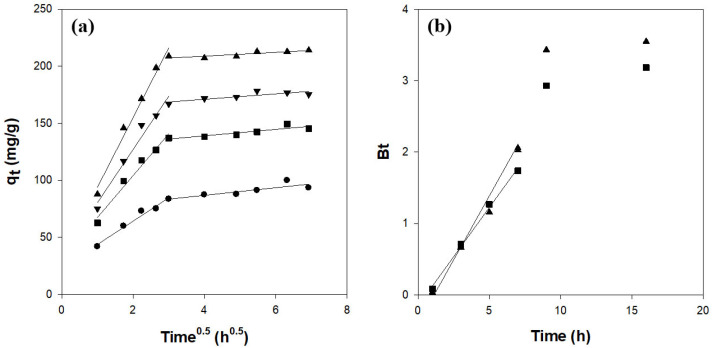
Diffusion kinetics for CV adsorption on κ-carrageenan/cellulose hydrogel beads: (**a**) intraparticle model and (**b**) Boyd’s model. Beads were incubated in a 50 mg/L CV solution (pH 7.0) at 25 °C. ●: CN1C6, ◼: CN2C5, ▼: CN3C4, ▲: CN4C3.

The adsorption of CV onto the hydrogel beads was analyzed in terms of multiple mass-transfer steps: transport of the dye through the bulk solution to the bead surface, diffusion across the boundary layer, intraparticle diffusion within the bead pores, and adsorption onto active sites. Under our experimental conditions, neither bulk transport nor surface adsorption was rate-limiting [39,40]. To evaluate the role of pore diffusion, an intraparticle diffusion model was applied, according to the following equation [38]:

(4) $$q_{t}=k_{id}t^{0.5}+C$$

where $k_{id}$ (mg/g/h^0.5^) is the intraparticle diffusion rate constant and C is the intercept reflecting boundary-layer effects. The intraparticle diffusion plots of $q_{t}$ vs. $t^{0.5}$ (Figure 6a) exhibited two distinct linear regions and did not pass through the origin. This indicates that intraparticle diffusion contributes to the overall rate but is not the sole rate-controlling step [40,41].

Boyd’s model was used to distinguish between film and pore diffusion in the following equation [38,41,42]:

(5) $$F=1-\frac{6}{\pi^{2}}exp\left(-Bt\right)$$

where *F* (= q_t_/q_e_) is the fractional attainment of equilibrium at time t and Bt is a function of *F*. This equation is summarized as follows:

(6) $$Bt=-0.4977-\ln\left(1-F\right)$$

The linear portion of the Bt vs. t plot (Figure 6b) does not intersect the origin, confirming that boundary-layer diffusion controls the initial adsorption stage and that intraparticle diffusion dominates at later stages [38,41,42].

#### 2.3.2. Isotherm Study of CV Adsorption on κ-Carrageenan/Cellulose Hydrogel Beads

**Figure 7:**
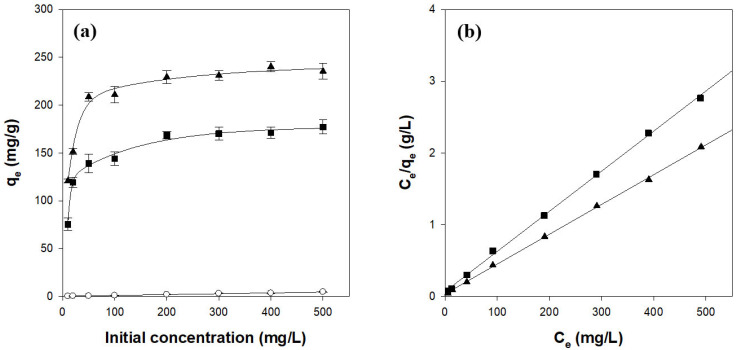
Isotherms (**a**) and Langmuir model fitting (**b**) for CV adsorption on κ-carrageenan/cellulose hydrogel beads. Beads were incubated in a CV solution (pH 7.0) at 25 °C for 24 h. ○: CN0C7, ◼: CN2C5, ▲: CN4C3.

The effect of the initial CV concentration on adsorption was evaluated over the range of 10–500 mg/L (Figure 7a). The amount of adsorbed CV increased sharply at low concentrations and leveled off above 50 mg/L, indicating saturation of the available binding sites. The data were fitted to the Langmuir isotherm, which was identified as the most appropriate model, assuming monolayer adsorption on a homogeneous surface [38]. The Langmuir equation can be linearized as follows:

(7) $$\;\frac{C_{e}}{q_{e}}=\frac{C_{e}}{q_{m}}+\frac{1}{q_{m}b}$$

where C_e_ (mg/L) is the dye concentration in the solution at equilibrium, q_e_ (mg/g) is the amount of dye adsorbed at equilibrium, q_m_ (mg/g) is the theoretical maximum adsorption capacity, and b (L/mg) is the Langmuir constant. As shown in Figure 7b and Table 3, the Langmuir model yielded excellent fits (r^2^ ≥ 0.997) and q_m_ values of 171.6 mg/g for CN2C5 and 240.5 mg/g for CN4C3, closely matching the experimental maxima of 175.7 mg/g and 238.1 mg/g, respectively. These results confirm that CV adsorption on κ-carrageenan/cellulose hydrogel beads proceeds predominantly through monolayer coverage and that increasing κ-carrageenan content enhances the maximum adsorption capacity [38,40].

The dimensionless separation factor (R_L_) signifies the favorability of adsorption, and the value of R_L_ indicates the shape of the adsorption isotherm. R_L_ can be derived from the parameters of the Langmuir model as follows:

(8) $$R_{L}=\frac{1}{1+bC_{0}}$$

where b (L/mg) is the Langmuir constant and C_0_ is the highest initial dye concentration (mg/L). An R_L_ value greater than 1 indicates unfavorable adsorption; R_L_ = 1 reflects a linear isotherm; R_L_ between 0 and 1 denotes favorable adsorption; and R_L_ = 0 corresponds to irreversible adsorption. For the CN2C5 and CN4C3 carrageenan/cellulose beads, R_L_ values of 0.01 and 0.02, respectively, were obtained, indicating that CV adsorption onto these hydrogel beads is highly favorable [38,43].

Our k-carrageenan/cellulose hydrogel beads (CN4C3) achieved a maximum CV uptake of 241 mg/g. This value exceeds the capacities of most reported κ-carrageenan-based adsorbents such as magnetic carrageenan/laponite RD at 164 mg/g and carrageenan/alginate montmorillonite composites at 89 mg/g (Table 4). Only κ-carrageenan/silver nanoparticle beads showed a slightly higher uptake (243 mg/g) [44]. These results show that a simple co-formulation with cellulose can match or surpass the performance of more complex modified materials. Notably, some of the reported materials with higher or comparable adsorption capacities utilize additional components bearing negative charges (e.g., laponite RD and alginate) or rely on ionic stabilization using K^+^ to maintain the carrageenan structure. In such systems, the native sulfate groups of carrageenan, which are critical functional groups for electrostatic binding with cationic dyes like CV, may be partially neutralized during gelation, thereby reducing their effective contribution to dye adsorption. In contrast, the CN4C3 beads developed in this study maintain structural integrity through hydrogen bonding with cellulose, without the need for ionic crosslinking. As a result, a greater number of sulfate groups remain accessible for dye interaction. This is supported by the observation that pure κ-carrageenan hydrogel beads stabilized with K^+^ exhibit a much lower q_m_ value of 52 mg/g [38], which corresponds to only about 20% of the CN4C3 value. These comparisons position our CN4C3 hydrogel beads among the top-performing κ-carrageenan-based adsorbents reported to date, particularly when considering the simplicity of preparation, absence of metal-ion crosslinking, and effective sulfate group exposure under mild conditions.

#### 2.3.3. Thermodynamic Study of CV Adsorption on κ-Carrageenan/Cellulose Hydrogel Beads

Thermodynamic parameters, which include standard free energy (∆G°), enthalpy change (∆H°), and entropy change (∆S°), are important factors to estimate the characteristics of the adsorption process. CV adsorption onto κ-carrageenan/cellulose hydrogel beads was performed at 25 °C, 35 °C, and 45 °C to obtain the thermodynamic parameters. The change in the thermodynamic parameters was calculated using the following equations [9]:

(9) ∆G°=∆H°−T∆S°

(10) $$∆G{}^{\circ}=-RT\ln K_{D}$$

The van’t Hoff equation can be derived from the previous equations and given as follows:

(11) $$\ln K_{D}=\frac{∆S{}^{\circ}}{R}-\frac{∆H{}^{\circ}}{RT}$$

(12) $$K_{D}=\frac{V}{m}\left(\frac{C_{0}}{C_{e}}-1\right)$$

where K_D_ (L/g) is the equilibrium constant, C_0_ (mg/L) is the initial concentration of the CV solution, C_e_ (mg/L) is the concentration of the CV solution at equilibrium, V (L) is the volume of adsorption, m (g) is the weight of the beads, R is the universal gas constant (8.314 J/mol/K), and T (K) is the temperature of the dye solution. The enthalpy and entropy change values were determined from the slope and intercept of Equation (11), and the results are presented in Table 5.

As shown in Table 5, all the thermodynamic parameters are negative. The ∆G° values are negative at all temperatures and become less negative as temperature increases, indicating that CV adsorption is spontaneous and that spontaneity decreases at higher temperatures [47]. The ΔG° values (−2.1 to −4.0 kJ/mol) indicate spontaneous and physical adsorption. Although pseudo-second-order kinetics are often linked to chemisorption, they can also arise from strong physical interactions like electrostatic attraction. Thus, the results suggest that site-specific physisorption governs the process [10,48]. The negative ΔH° value confirms that the adsorption process is exothermic and likely governed by weak, non-covalent interactions. Meanwhile, the negative ΔS° value suggests decreased randomness at the solid–liquid interface due to restricted movement of dye molecules after adsorption [10,47].

#### 2.3.4. Effect of pH on CV Adsorption by κ-Carrageenan/Cellulose Hydrogel Beads

**Figure 8:**
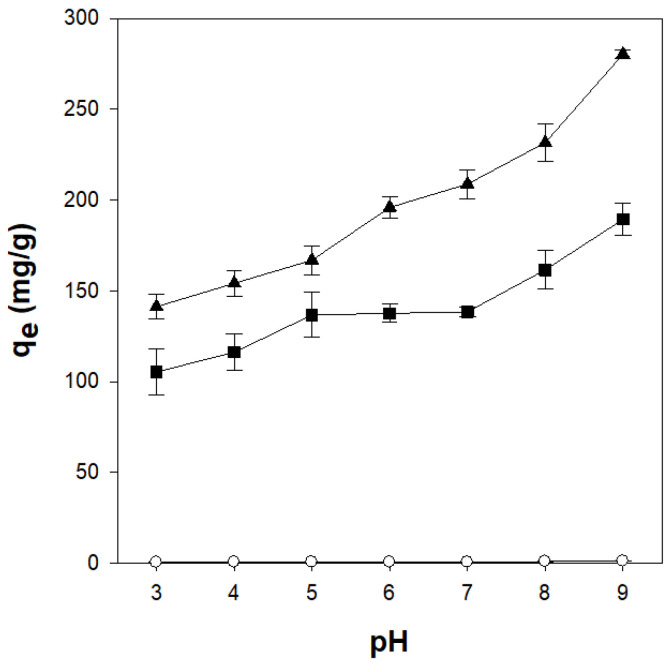
Effect of pH on crystal adsorption by κ-carrageenan/cellulose hydrogel beads. Beads were incubated in a 50 mg/L CV solution at 25 °C for 24 h. ○: CN0C7, ◼: CN2C5, ▲: CN4C3.

The pH of the dye solution is one of the factors affecting the adsorption process because the pH of the solution can alter the active adsorption sites on the adsorbent. To confirm the influence of the pH of the dye solution on the adsorption process, CV adsorption was analyzed at pH values ranging from 3 to 9 (Figure 8). The amount of CV adsorbed onto the κ-carrageenan/cellulose hydrogel beads increased as the pH was increased from 3 to 9. This behavior is attributed to enhanced electrostatic attraction between the negatively charged sulfate groups in carrageenan and the cationic CV molecules [49]. In the acidic solution, the high concentration and small molecular size of H^+^ allowed it to compete with CV. As a result, protonation of the sulfate groups under acidic conditions reduced the electrostatic attraction, leading to a lower adsorption capacity. In addition, the reduction in negative surface charge due to protonation diminished the electrostatic attraction toward CV, further lowering the adsorption efficiency [50]. Generally, pure κ-carrageenan gel becomes weakened and eventually dissolves under acidic conditions below pH 4.3. However, the κ-carrageenan/cellulose beads (CN2C5 and CN4C3) developed in this study maintained both their CV adsorption capacity and gel structure even at pH 3. This improved acid stability is presumably due to strong hydrogen bonding between carrageenan and cellulose rather than the conventional stabilization of carrageenan gels by ionic interactions with K^+^. As shown in Figure S3, pure κ-carrageenan beads disintegrated within 10 min at pH 3, while CN4C3 beads maintained their shape for at least 60 min. This suggests that cellulose incorporation significantly improves acid stability without relying on metal-ion crosslinking.

### 2.4. Reusability of κ-Carrageenan/Cellulose Hydrogel Beads

**Figure 9:**
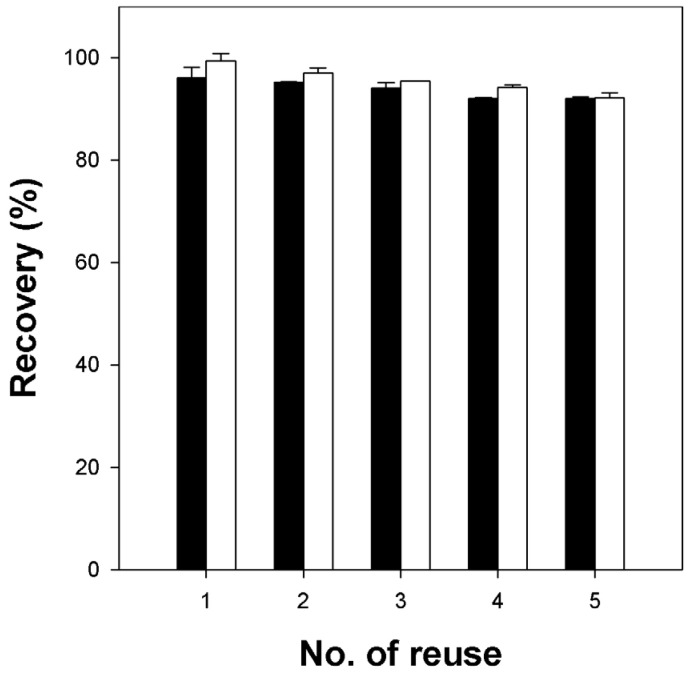
Reusability of κ-carrageenan/cellulose hydrogel beads (CN4C3) for CV removal. Black bars represent desorption recovery, calculated as the amount of dye desorbed in each cycle divided by the amount adsorbed upon first use and multiplied by 100 to obtain a percentage (%). White bars represent adsorption recovery, calculated as the amount of dye re-adsorbed in each cycle divided by the amount adsorbed upon first use and multiplied by 100 to obtain a percentage (%).

The reusability of carrageenan/cellulose hydrogel beads (CN4C3) was evaluated by measuring CV desorption in a water/ethanol (50/50 *v*/*v*) solution containing 0.5 M potassium chloride (KCl), followed by re-adsorption using the regenerated beads. As shown in Figure 9, both the adsorption and desorption recoveries decreased slightly over multiple reuse cycles. However, both recoveries consistently remained above approximately 90% of their initial values, revealing good reusability and stability of the κ-carrageenan/cellulose hydrogel beads for repeated dye adsorption–desorption cycles. Additionally, morphological observations (Figure S4) confirmed that CN4C3 beads retained their shape and functionality throughout the reuse cycles, unlike pure κ-carrageenan beads, which showed significant deformation.

## 3. Conclusions

In this study, κ-carrageenan/cellulose hydrogel beads were successfully prepared using an ionic liquid ([Emim][Ac]) mixed with DMF as a co-solvent, thereby presenting an effective method for removing cationic dyes from wastewater. The hydrogel beads were synthesized through an aqueous ethanol-induced phase separation method, eliminating the need for metal-ion crosslinkers and producing robust beads with an interpenetrating network structure. This novel formulation strategically combined the advantageous properties of cellulose (mechanical strength and structural integrity) with those of κ-carrageenan (highly hydrophilic and anionic sulfate groups), significantly enhancing the cationic dye adsorption capacity. Although the incorporation of κ-carrageenan significantly increased the initial swelling ratio of the beads, re-swelling was markedly reduced after drying due to irreversible network collapse. Nevertheless, the beads maintained a substantial CV adsorption capacity, likely because accessible sulfate groups remained concentrated near the surface, even after structural densification. These adsorption characteristics were further confirmed through detailed experimental analyses. The experimental results show that increasing the κ-carrageenan content proportionally improves the adsorption performance of the hydrogel beads, with the highest adsorption capacity reaching 241 mg/g for CV. The adsorption kinetics aligned well with the pseudo-second-order kinetic model, indicating chemisorption as the predominant mechanism. The intraparticle diffusion and boundary-layer diffusion models provided insights into the adsorption process dynamics. Isotherm analyses revealed that CV adsorption on these hydrogel beads strongly conformed to the Langmuir isotherm, which is indicative of monolayer adsorption on homogeneous surfaces. The thermodynamic assessments indicated that the adsorption process was spontaneous, exothermic, and primarily governed by physical interactions. Furthermore, the hydrogel beads exhibited stable adsorption capacity across a wide pH range (pH 3–9), suggesting their applicability in diverse wastewater conditions without structural degradation or loss of performance. In addition, the beads maintained a robust adsorption performance and structural integrity over multiple reuse cycles, highlighting their excellent reusability and stability.

Overall, the κ-carrageenan/cellulose hydrogel beads developed in this study represent a promising, cost-effective, and environmentally friendly solution for treating wastewater containing cationic dyes. Future research should aim to optimize the bead composition further and assess their adsorption capacity for a wider range of pollutants, including heavy metal ions, to enhance their potential for industrial application. Although this study employed organic co-solvents such as DMF to facilitate hydrogel formation, future work will explore greener alternatives, such as deep eutectic solvents (DES), to further improve the sustainability of the preparation process.

## 4. Materials and Methods

### 4.1. Materials

Cellulose (cotton linter with long fiber form), κ-carrageenan, [Emim][Ac] (purity > 95% by HPLC), and DMF (anhydrous > 99.8%) were purchased from Sigma-Aldrich (St. Louis, MO, USA). Crystal violet (CV), ethyl alcohol, and potassium chloride were purchased from Samchun Pure Chemicals (Pyeongtaek-si, Republic of Korea). All other chemicals used in this study were of analytical grade and used without further purification.

### 4.2. Preparation of κ-Carrageenan/Cellulose Hydrogel Beads

**Figure 10:**
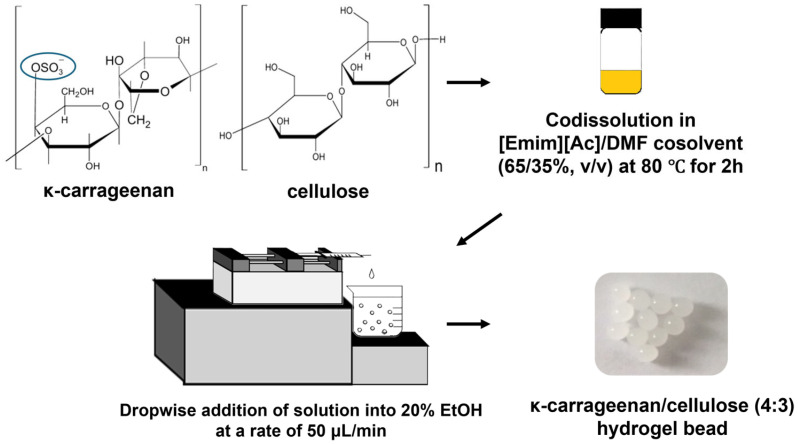
Process scheme for the preparation of κ-carrageenan/cellulose hydrogel beads using an ionic liquid mixture.

κ-Carrageenan/cellulose hydrogel beads were prepared using a previously reported method with slight modifications [27,30]. Various amounts of κ-carrageenan and cellulose (total content of 7% *w*/*v*) were dissolved in a mixture of [Emim][Ac]/DMF (65/35% *v*/*v*) by stirring at 80 °C for 2 h. Transparent solutions containing κ-carrageenan and cellulose were then dried under vacuum at 60 °C to remove air bubbles. One milliliter of the κ-carrageenan/cellulose solutions was added drop-wise using a 1 mL plastic syringe with a 26-gauge needle and a syringe pump (LSP01-2A; Longer Pump; Baoding, China) into 200 mL of 20% ethanol/water at a rate of 50 μL/min with vigorous stirring. The hydrogel beads were then cured in 20% ethanol/water for 20 min. After reconstitution, the hydrogel beads were washed with distilled water and stirred overnight (Figure 10).

### 4.3. Characterization of κ-Carrageenan/Cellulose Hydrogel Beads

A Vernier caliper was used to calculate the wet bead size of the hydrogel beads, and the values were averaged. The diameter of each bead is measured at three angles. To determine the dry bead weight, 40 hydrogel beads were dried at 60 °C for 24 h. The surfaces of the hydrogel beads were analyzed using scanning electron microscopy (SEM; AURIGA; Jena, Germany). The hydrogel beads were frozen at −80 °C and then dried overnight at −80 °C under a vacuum. All the samples were sputter-coated with gold.

### 4.4. Swelling Ratio of κ-Carrageenan/Cellulose Hydrogel Beads

Forty beads were incubated in distilled water for 48 h at room temperature to determine the initial swelling ratio of freshly prepared κ-carrageenan/cellulose hydrogel beads. The swollen beads were gently blotted with Kimwipes to remove excess surface water and then weighed. Subsequently, the dry weights of the beads were measured after drying at 60 °C for 24 h. The initial swelling ratio (g/g dry weight) was calculated as follows:

(13) $$Initial\;swelling\;ratio=\frac{W_{initial}-W_{d}}{W_{d}}$$

where $W_{initial}$ and $W_{d}$ are the weights of freshly prepared hydrogel beads in the swollen state and after complete drying, respectively [33].

The re-swelling ratio was measured using beads dried at 60 °C for 24 h. Forty dried beads were weighed and then incubated in distilled water at room temperature for 48 h. The re-swollen beads were gently blotted with Kimwipes to remove excess water and weighed again. The re-swelling ratio (g/g dry weight) was calculated using the following equation:

(14) $$Reswelling\;ratio=\frac{W_{reswell}-W_{d}}{W_{d}}$$

where $W_{reswell}$ and $W_{d}$ are the weights of the dried beads after re-swelling and complete drying, respectively.

### 4.5. Dye Adsorption on κ-Carrageenan/Cellulose Hydrogel Beads

Experiments to study the adsorption of CV onto κ-carrageenan/cellulose hydrogel beads were performed under continuous shaking at 120 rpm in a water bath. Three hydrogel beads were added to 20 mL of CV solution (with pH adjusted as necessary) in 50 mL conical tubes and incubated at 25 °C. The concentration of CV remaining in solution was determined spectrophotometrically at 590 nm using an Optizen 3220UV UV-vis spectrophotometer (Mecasys Co., Ltd., Daejeon, Republic of Korea).

To investigate the adsorption kinetics, aliquots (100 μL) of the CV solution (initial concentration of 50 mg/L, pH 7) were periodically removed, diluted with distilled water, and analyzed. The amount of CV adsorbed onto κ-carrageenan/cellulose hydrogel beads at time t ($q_{t}$, mg/g) was calculated using the following equation:

(15) $$q_{t}=\frac{\left(C_{0}-C_{t}\right)}{m}\times V$$

where $C_{0}$ (mg/L) is the initial concentration of CV, $C_{t}$ (mg/L) is the concentration of CV at time t, m (g) is the total dry weight of the beads, and V (L) is the volume of the adsorption solution.

For the isotherm studies, adsorption experiments were conducted using various initial CV concentrations (10, 20, 50, 100, 200, 300, 400, and 500 mg/L) under batch conditions for 24 h. For the thermodynamic analyses, adsorption experiments were performed at temperatures of 35 and 45 °C. The equilibrium adsorption capacity ($q_{e}$, mg/g) was calculated as follows:

(16) $$q_{e}=\frac{\left(C_{0}-C_{e}\right)}{m}\times V$$

where $C_{e}$ (mg/L) is the equilibrium concentration of CV in solution.

The effect of solution pH on adsorption was evaluated by adjusting the initial pH of the CV solution to values ranging from 3 to 9 using 0.1 M HCl or NaOH and measuring the equilibrium adsorption capacity ($q_{e}$) at each pH condition.

All adsorption experiments were performed in triplicate, and the results were averaged.

### 4.6. Dye Desorption and Bead Reusability

To evaluate the desorption of CV from κ-carrageenan/cellulose hydrogel beads (CN4C3), a desorption solution consisting of 0.5 M KCl in a 50:50 (*v*/*v*) water/ethanol mixture was used. After the initial adsorption, the dye-loaded beads were immersed in 20 mL of the desorption solution and agitated in a shaking water bath at 120 rpm and 25 °C for 3 h. The concentration of desorbed CV was measured at 590 nm using a spectrophotometer and quantified using a calibration curve prepared in the same desorption medium.

Following desorption, the beads were retrieved, thoroughly rinsed with distilled water to remove the residual desorption solution, and reused for subsequent adsorption cycles. Re-adsorption was performed under identical conditions, as described in Section 4.5, by exposing the regenerated beads to a 50 mg/L CV solution (pH 7) at 25 °C for 24 h. This adsorption–desorption cycle was repeated five times to assess the reusability and stability of the hydrogel beads. The reusability test was conducted in triplicate.

## Figures and Tables

**Table 1:** Wet sizes and dried weights of prepared κ-carrageenan/cellulose hydrogel beads.

Sample Abbreviation	Carrageenan/Cellulose * (%/%)	Average Wet Bead Size (mm)	Average Dried Weight of Bead (mg/bead)
CN0C7	0/7	2.20 ± 0.02	0.57 ± 0.01
CN1C6	1/6	2.30 ± 0.04	0.48 ± 0.02
CN2C5	2/5	2.40 ± 0.04	0.40 ± 0.02
CN3C4	3/4	2.45 ± 0.02	0.35 ± 0.00
CN4C3	4/3	2.53 ± 0.03	0.25 ± 0.01

* Content in gel-forming solution (65/35% *v/v* [Emim][Ac]/DMF).

**Table 2:** Kinetic parameters for CV adsorption on k-carrageenan/cellulose hydrogel beads.

Sample	Pseudo-Second-Order Model			q_e, exp_ (mg/g)
	k_2_ (×10^−3^ g/mg/h)	q_e, cal_ (mg/g)	r^2^	
CN1C6	5.3	99.0	0.997	90.3
CN2C5	4.8	150.8	0.999	141.7
CN3C4	4.8	181.5	1.000	175.3
CN4C3	4.0	219.5	1.000	212.2

**Table 3:** Isotherm parameters for CV adsorption on κ-carrageenan/cellulose hydrogel beads.

Sample	Langmuir Model			q_m, exp_ (mg/g)
	b (×10^−3^ L/mg)	q_m_ (mg/g)	r^2^	
CN2C5	143.5	171.6	0.997	175.7
CN4C3	122.3	240.5	1.000	238.1

**Table 4:** Maximum adsorption capacity of various adsorbents containing k-carrageenan for CV.

Adsorbent	q_m_ (mg/g)	Ref.
Magnetic carrageenan-g-poly(methacrylic acid) nanocomposite	28	[32]
Carrageenan wet beads	52	[38]
Carrageenan-poly(acrylamide)/sodium montmorillonite nanocomposite	43	[45]
Carrageenan-g-poly(acrylamide)/sepiolite nanocomposite hydrogels	47	[21]
Oxidized multiwalled carbon nanotube/carrageenan/Fe_3_O_4_ composite	47	[40]
Carrageenan/laponite RD	80	[42]
Magnetic carrageenan beads	85	[17]
Carrageenan/alginate/sodium montmorillonite nanocomposite hydrogels	89	[9]
Carrageenan/poly(vinyl alcohol) hydrogels	121	[46]
Magnetic carrageenan/laponite RD	164	[10]
Carrageenan/silver nanoparticles beads	243	[44]
Carrageenan/cellulose hydrogel beads	241	This work

**Table 5:** Thermodynamic parameters for CV adsorption on k-carrageenan/cellulose hydrogel beads.

Sample	T (K)	ΔG° (kJ/mol)	ΔS° (J/K/mol)	ΔH° (kJ/mol)	r^2^
CN2C5	298.15	−3.0	−39.4	−14.7	0.911
	308.15	−2.3			
	318.15	−2.1			
CN4C3	298.15	−4.0	−54.7	−20.2	0.870
	308.15	−3.0			
	318.15	−2.8			

## Data Availability

The original contributions presented in this study are included in the article/Supplementary Materials. Further inquiries can be directed to the corresponding author.

## Supplementary Materials

The following supporting information can be downloaded at: https://www.mdpi.com/article/10.3390/gels11080596/s1, Figure S1: Visible spectra in the 400–1000 nm range of films composed of pure cellulose (CN0C7), 2%/5% κ-carrageenan/cellulose (CN2C5), and 4%/3% κ-carrageenan/cellulose (CN4C3); Figure S2: FT-IR spectra of the CN4C3 film: (a) before CV adsorption and (b) after CV adsorption; Figure S3: Time-dependent morphological changes in CN4C3 and κ-carrageenan hydrogel beads in pH 3 solution at 25 °C. The pure κ-carrageenan hydrogel beads were prepared using K^+^ ions as crosslinkers according to a previously reported method; Figure S4: Morphological stability and reusability of κ-carrageenan-based beads under repeated use: (a) fatigue test comparing the morphological changes in pure κ-carrageenan hydrogel beads (bottom row) and CN4C3 hydrogel beads (top row) over five cycles of reuse, (b) reusability test of CN4C3 hydrogel beads during five consecutive cycles of crystal violet adsorption (top row) and desorption (bottom row).

## Abbreviations

The following abbreviations are used in this manuscript:

3D	three-dimensional
[Emim][Ac]	1-ethyl-3-methylimidazolium acetate
CV	crystal violet
DMF	N,N-dimethylforamide
DMSO	dimethyl sulfoxide
SEM	scanning electron microscopy
EtOH	ethanol
KCl	potassium chloride
q_e_	adsorption capacity at equilibrium
q_t_	adsorption capacity at time t
C_0_	concentration of dye at initial
C_e_	concentration of dye at equilibrium
C_t_	concentration of dye at time t
R_L_	dimensionless separation factor
T	temperature
R	gas constant
V	volume of solution
m	mass of adsorbent
*X*_CN_	carrageenan mass fraction in the bead
CN0C7	κ-carrageenan/cellulose (0:7) hydrogel bead
CN1C6	κ-carrageenan/cellulose (1:6) hydrogel bead
CN2C5	κ-carrageenan/cellulose (2:5) hydrogel bead
CN3C4	κ-carrageenan/cellulose (3:4) hydrogel bead
CN4C3	κ-carrageenan/cellulose (4:3) hydrogel bead
