# Supplementary material for: Metal-Ion-Free Preparation of κ-Carrageenan/Cellulose Hydrogel Beads Using an Ionic Liquid Mixture for Effective Cationic Dye Removal
Source: Gels. 2025 Aug 1;11(8):596. doi: 10.3390/gels11080596 (PMC12385765; doi:10.3390/gels11080596)
Supplement: Supplementary file 1 [file gels-11-00596-s001.zip › gels-3759181-supplementary.pdf]

Electronic Supplementary Information

Metal-Ion-Free Preparation of κ-Carrageenan/Cellulose Hydrogel Beads Using an Ionic Liquid Mixture for Effective Cationic Dye Removal

Dojin Kim [1,†], Dong Han Kim [1,†], Jeong Eun Cha [1], Saerom Park [2,*] and Sang Hyun Lee [1,2,*]

[1] Advanced Materials Program, Department of Biological Engineering, Konkuk University, Seoul 05029, Republic of Korea

[2] Department of Biological Engineering, Konkuk University, Seoul 05029, Republic of Korea

[*] Correspondence: angel4y@naver.com (S.P.); sanghlee@konkuk.ac.kr (S.H.L.); Tel.: +82-10-4038-4656 (S.P.); +82-2-2049-6269 (S.H.L.)

[†] These authors contributed equally to this work.

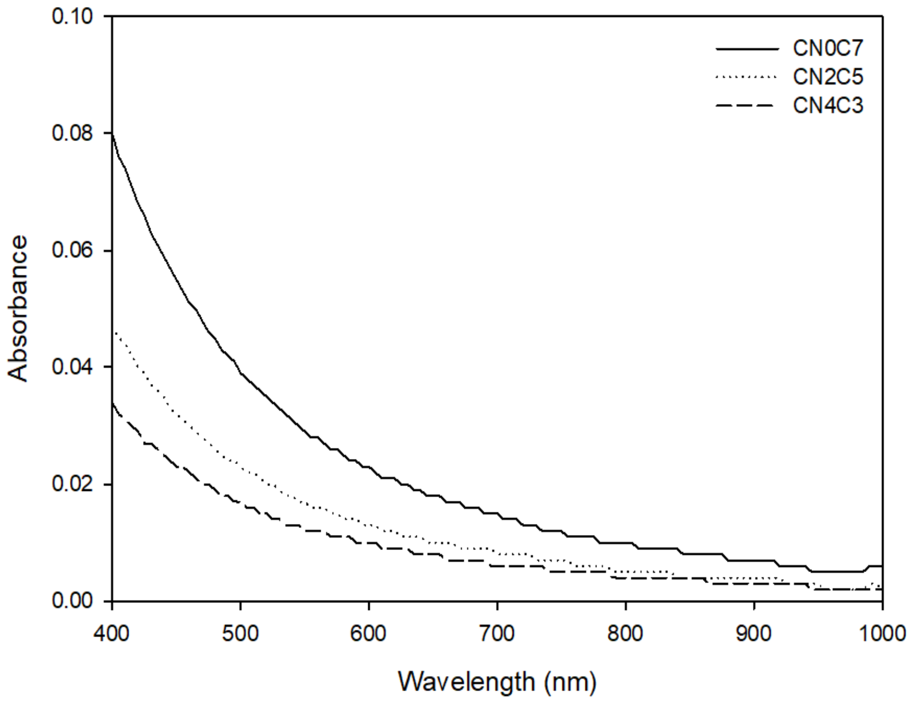

Figure S1. Visible spectra in the 400-1000 nm range of films composed of pure cellulose (CN0C7), 2%/5% κ-carrageenan/cellulose (CN2C5), and 4%/3% κ-carrageenan/cellulose (CN4C3).

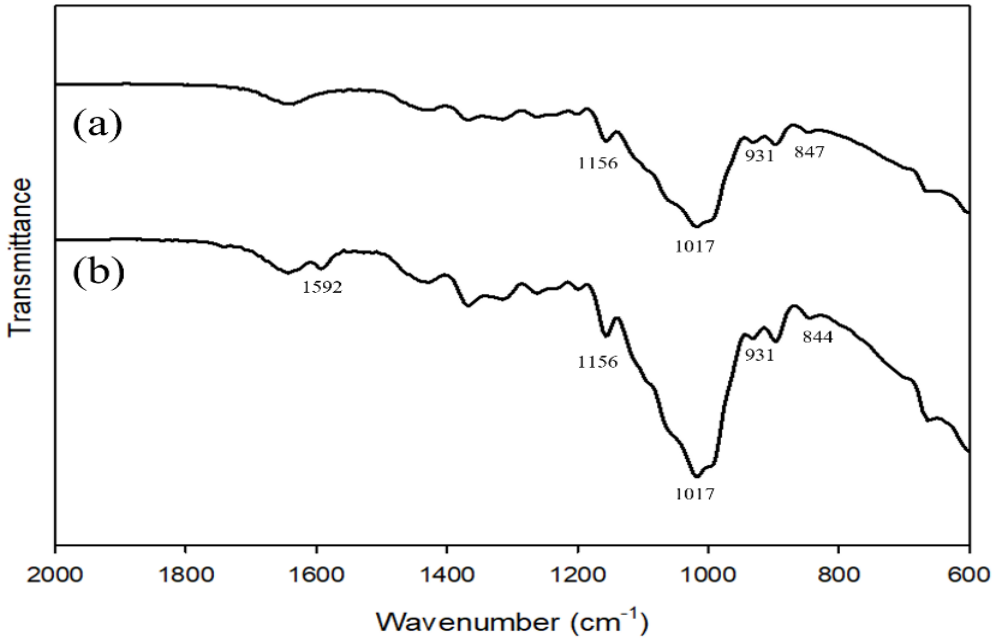

Figure S2. FT-IR spectra of the CN4C3 film: (a) before CV adsorption and (b) after CV adsorption.

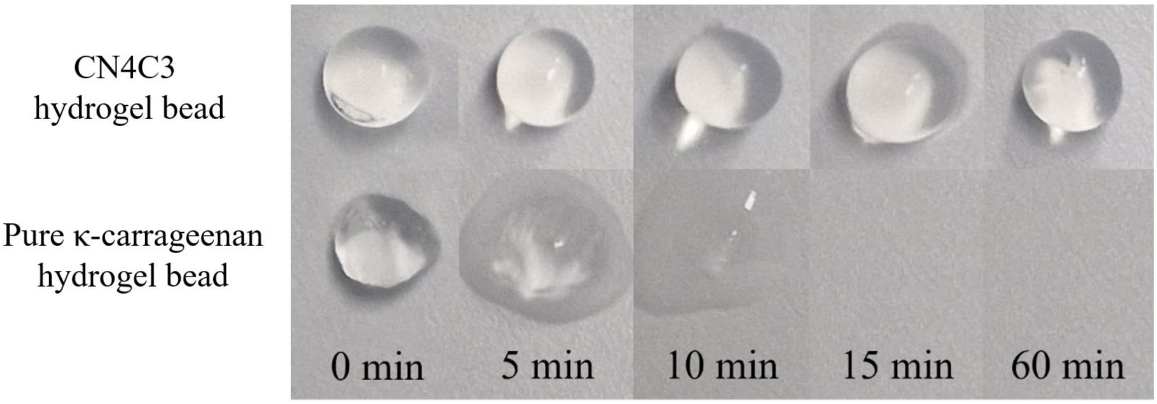

Figure S3. Time-dependent morphological changes of CN4C3 and κ-carrageenan hydrogel beads in pH 3 solution at 25℃. The pure κ-carrageenan hydrogel bead was prepared using K^+ ions as cross-linkers according to a previously reported method [38].

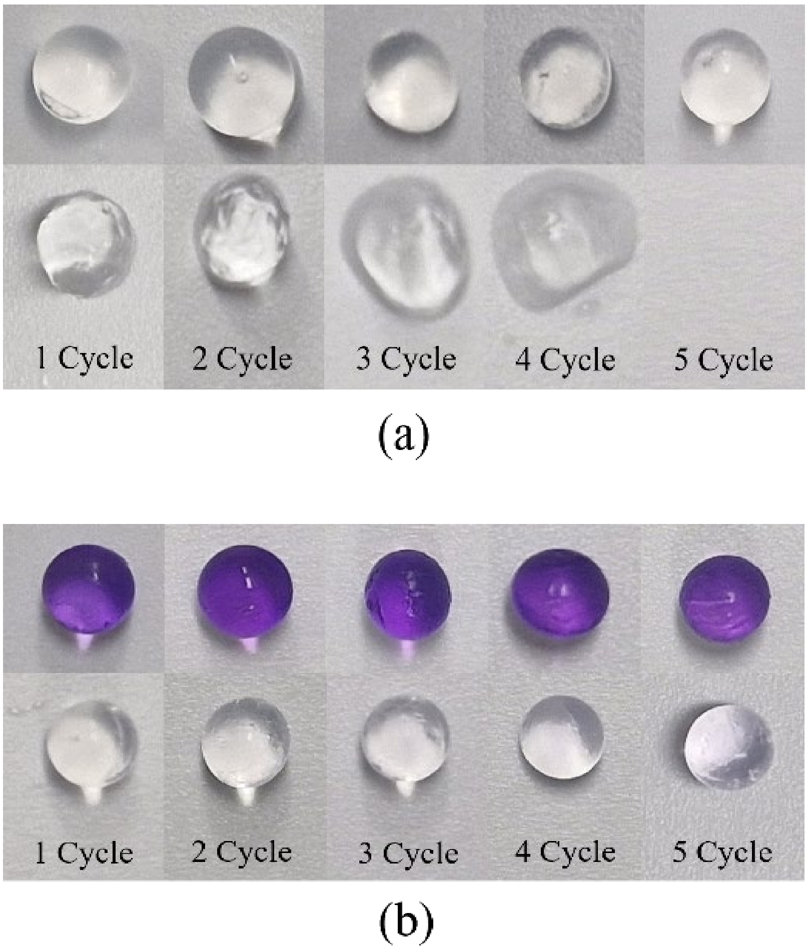

Figure S4. Morphological stability and reusability of κ-carrageenan-based beads under repeated use: (a) Fatigue test comparing the morphological changes of hydrogel beads composed of pure κ-carrageenan hydrogel bead [38] (bottom row) and CN4C3 hydrogel bead (top row) over five cycles of reuse, (b) Reusability test of CN4C3 hydrogel bead during five consecutive cycles of crystal violet adsorption (top row) and desorption (bottom row).
